# Do solidarity and reciprocity obligations compel African researchers to feedback individual genetic results in genomics research?

**DOI:** 10.1186/s12910-020-00549-4

**Published:** 2020-11-04

**Authors:** Dimpho Ralefala, Mary Kasule, Ambroise Wonkam, Mogomotsi Matshaba, Jantina de Vries

**Affiliations:** 1 Department of Medicine, Faculty of Health Sciences, University of Cape Town, Cape Town, 7925 South Africa; 2 Office of Research and Development, University of Botswana, Gaborone, Botswana; 3 Deputy Dean’s Office, Faculty of Health Sciences, Groote Schuur Hospital, Cape Town, South Africa; 4 Botswana-Baylor Children’s Clinical Centre of Excellence, Gaborone, Botswana; 5 Baylor College of Medicine, Houston, TX USA

**Keywords:** Solidarity, Reciprocity, Feedback, Individual, Genetic research results, Genomics research, Botswana, Africa

## Abstract

**Background:**

A key ethical question in genomics research relates to whether individual genetic research results should be disclosed to research participants and if so, which results are to be disclosed, by whom and when. Whilst this issue has received only scarce attention in African bioethics discourse, the extension of genomics research to the African continent has brought it into sharp focus.

**Methods:**

In this qualitative study, we examined the views of adolescents, parents and caregivers participating in a paediatric and adolescent HIV-TB genomic study in Botswana on how solidarity and reciprocity obligations could guide decisions about feedback of individual genetic research results. Data were collected using deliberative focus group discussions and in-depth interviews.

**Results:**

Findings from 93 participants (44 adolescents and 49 parents and caregivers) demonstrated the importance of considering solidarity and reciprocity obligations in decisions about the return of individual genetic research results to participants. Participants viewed research participation as a mutual relationship and expressed that return of research results would be one way in which research participation could be reciprocated. They noted that when reciprocity obligations are respected, participants feel valued and not respecting reciprocity expectations could undermine participant trust and participation in future studies.

**Conclusions:**

We conclude that expectations of solidarity and reciprocity could translate into an obligation to feedback selected individual genetic research results in African genomics research.

## Background

Feedback of individual genetic research results to research participants has increasingly become a topic of debate in bioethics, not in the least because such results may be relevant to the health of participants and their families [1]. Whilst some researchers have expressed a concern that feeding back individual genetic research results contravenes the traditional goal of producing generalizable knowledge for the good of society [2], there appears to be a growing consensus regarding both moral and legal obligations of researchers, to feedback particular results [3–5]. Key values that are discussed in this literature are respect, autonomy, charity, mutuality, and reciprocity [6].

Amongst the plethora of papers that have commented on issues relating to the feedback of individual genetic results in genomics research, only a small subset is specific to low and middle-income countries (LMICs). Of those, Kerasidou’s paper [7] seems to engage most with the peculiarities of the LMIC research context, yet it focuses on the return of aggregate and not individual findings. The others [8–11] seem to largely frame their work in terms of the poverty and vulnerability of African research participants. Richardson and Cho [12] elaborate an entrustment model for the return of results, in which they premise an obligation to return results on the inability of participants to afford or access healthcare through other means. As such, this account is premised on participants’ vulnerability, with the vulnerability arising out of poverty*.* Ortiz-Osorno and colleagues [8] limit their discussion of feedback of individual clinical results to a question of actionability, with specific focus on the inability of some research participants to afford the healthcare that the individual results would mandate. Similar to Richardson, this work is also largely premised on the poverty and potential vulnerability of the LMIC research participants taking part in research. Finally, MacKay [10] advances a “do the most good” principle to guide decisions about the return of research results and specifically ties this notion to the perceived poverty and vulnerability of African research participants. Of note is that all of these accounts seem to be specific to genomics research that is led by principal investigators based in high-income countries (HICs) where the main question is whether and to what extent HIC researchers have an obligation to return results to participants in poorer countries [8–10, 12].

The focus on the poverty and vulnerability of African research participants has two effects. First, it categorically treats all Africans as poor or vulnerable and of course the reality is not that simple. Second, and perhaps more importantly, it tends to disregard some of the fundamental ethical norms that guide Africans’ way of life, as it does the life of others, namely solidarity and reciprocity. Although there is a fledging literature that advances reciprocity arguments with regards to feedback of results in genomics research, this issue is often mentioned in passing and neither elaborated nor contextualised to the African setting [7, 13, 14]. Richardson and Cho [12] briefly explain reciprocity as an attribute of relationships which involves an exchange of burdens and benefits. Bredenoord and colleagues [13, 14] state that even though participants often participate in research for altruistic or other reasons, they may anticipate a reward for instance in the form of feedback of individual genetic research results. Overall, in this literature, reciprocity seems to be advanced merely as a way of appreciating research participants or showing gratitude for their participation in research. Importantly, the discretion to appreciate participants seems to mostly lie with the researchers. This differs with accounts of reciprocity in the African research context, where it is not only seen as a moral obligation but a fundamental social norm where deviance has a consequence.

The concepts of solidarity and reciprocity in African societies are grounded in the principle of Ubuntu or Botho, as commonly known in Setswana. According to Saule as cited by Mufune [15], this “*emphasizes the principle of helping others as a way of helping oneself, collective activity and well-being rather than individualism, unification rather than division, respect for elders and sharing*” (p. 21). Although some of these themes like a sense of community may also be present elsewhere, the dominant philosophies there do not generally consider these values as fundamental in prescribing obligations, as is the case in Ubuntu philosophy [16]. As highlighted by Metz [17], the communal nature of the African way of life which is centred around Ubuntu, is fundamental in defining personhood, dictates ethical obligations, and requires members of the community to identify with others as well as demonstrate solidarity towards each other. In this context, solidarity is defined as a reciprocal relationship in which members of a community who recognise their similarities, including their shared vulnerabilities and that they are dependent on one another, decide to collaborate with each other with the goal of avoiding suffering, reducing health inequalities and ensuring that all parties flourish [18]. This form of solidarity is not directed by sympathy for the disadvantaged. Instead, people in solidarity treat each other as equals in a balanced relationship, especially in relation to the shared interest, goal or situation [19]. Solidarity and reciprocity are interconnected, for solidarity to grow there must be a certain level of reciprocity [20]. Therefore, honouring reciprocity obligations is seen as one way of showing Ubuntu and solidarity and is of key importance in maintaining societal relationships and stability in the African context.

Yet although the concept of solidarity has steadily gained more attention in bioethics literature [19–26], again few papers addresses this issue in research in LMICs [18, 27]. No empirical study has explored how these two concepts that are integral to the African way of life, could impact on African researchers’ obligations to feedback individual genetic research results. In our study, we were interested in understanding better how solidarity and reciprocity feature in relation to discussions around the feedback of individual genetic research results particularly from the view of participants in an African genomic research study.

## Methods

The work presented in this paper was part of a larger study aimed at exploring expectations and preferences for feedback of individual genetic research results with parents and caregivers of participants and adolescents involved in an HIV-TB genomics research in Botswana.

### Study setting and population

This study was conducted as part of the Individual Findings in Genetics Research in Africa (IFGENERA), a collaborative project between the University of Cape Town, University of Botswana and Botswana-Baylor Children’s Clinical Centre of Excellence (BBCCCE). The study was based in Botswana, a country with an estimated population size of just over 2 million people, majority of which are Christians (86.7%) with 63.9% living in urban areas (cities, towns and urban villages) as of 2017 statistics [28]. The country’s adult literacy rate stood at 90% in 2014 [29]. For many years, Botswana has been challenged with one of the worst HIV/AIDS burdens in the world with an estimated prevalence of 18.5% [30] coupled with a high HIV/TB co-infection estimated at 60% [31].

As a result of this HIV/TB burden, the Collaborative African Genomics Network (CAfGEN) conducted at BBCCCE, was initiated to study genes of children with HIV and TB, to inform the development of new therapies to prevent or supress these infections [32]. The CAfGEN study is a collaborative project within the Human Heredity and Health for Africa (H3Africa) Consortium [33] and uses genomics approaches to identify host genetic factors that are important for the progression of HIV and HIV-TB infection in paediatric and adolescent African populations [34]. This genomic study was selected as a case study because it is amongst the few genomic studies being conducted in Botswana and offered an opportunity to explore questions around the feedback of individual results in a genomics research. The adolescent cohort in this study provided an opportunity to explore perspectives of adolescents regarding these issues, as well as of the parents of younger children.

### Data collection

Participants of this study were purposively selected. Recruitment of participants was done face-to-face during participants’ clinic visits at the BBCCCE as well as telephonically where necessary. This study targeted (1) parents and caregivers of children (aged 2–18 years) involved in the CAfGEN study and (2) adolescents (aged 15–18 years) involved in the same study. BBCCCE is a clinical centre that provides HIV/AIDS treatment and support to children, adolescents and their families. As a result, all of the adolescents who participated in the CAfGEN study, who were also recruited into our study, received care from BBCCCE.

**Fig. 1:**
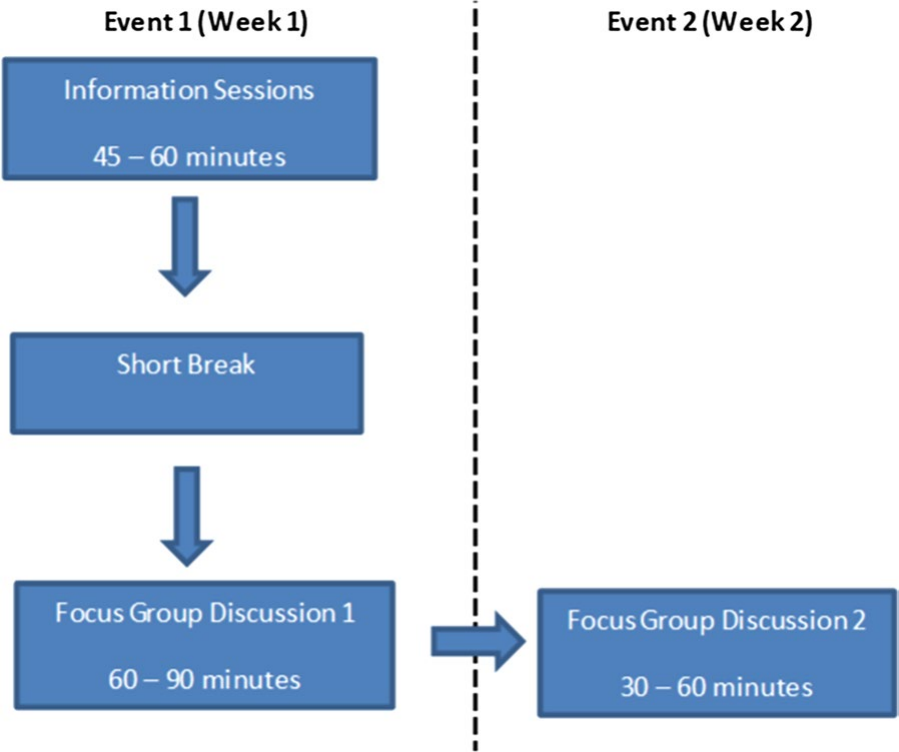
Overview of research process

We used a qualitative study methodology involving deliberative focus group discussions (dFGDs) [35] and in-depth interviews (IDIs). Deliberative Focus Groups combine a traditional FGD approach with extended opportunities for learning and discussion, in an attempt at allowing participants to engage with the study topic in more depth. A dFGD manual with step by step instructions for the dFGD process was developed after a rigorous literature review (see Additional file 1). The tool was then piloted with 3 groups of participants (2 groups of parents and caregivers and 1 group of adolescents) to ensure content validity and practicability, after which it was used for the main study. The data was collected from February 2019 up to March 2020. The dFGD technique adopted for this study involved an interactive information sharing session with participants during the initial meeting, where the provision of information was iterated with discussion of a few scenarios. Participants were provided with information about genes and what they are, how they work, what happens when there are problems with genes, how genes impact on health as well as what genomics research is. This information session took about 45–60 min (see Fig. 1 for a schematic overview).

DFGDs for parents and caregivers were held separate from adolescents dFGDs, although in some instances we recruited pairs of parent/children who participated in separate events. While these participants may have discussed the dFGD questions between themselves at home, and possibly influencing each other’s views, we could not rule out that other participants who were not paired did not do so, as both groups were encouraged to discuss issues raised in the study with family members between the 1st meeting and the 2nd meeting, which were held a week apart. No parent/children pairs were recruited for the IDIs.

Holding the dFGDs in two separate stages was intended to: (1) create enough time for engagement with participants and support information sharing especially because we were investigating a topic with which participants were unfamiliar; (2) provide participants with an opportunity to reflect on the issue being studied in-between the discussions; and (3) allow revisiting of views over time [36, 37]. Whilst we interrogated a broader range of issues relating to the return of individual genetic research results, we also specifically probed solidarity and reciprocity, for instance in relation to questions about why participants thought that individual genetic results should be returned, whether, how and why participants should be appreciated for their participation and who they thought was responsible for covering costs of follow-up care and why.

Transcription of the dFGD data was done on a rolling basis. A topic guide was developed for the IDIs (see Additional file 2) after completing 12 dFGD events (6 groups) and the remaining 12 dFGD events followed as per the dFGD manual. The IDIs were conducted after completion of the dFGD data collection, to ensure that in-depth data about areas of controversy was collected. The IDI topic guide was piloted with 5 participants (3 parents/caregivers and 2 adolescents). Although we did not have a strict criteria to assess this, we mostly selected participants who either held uncommon views or who engaged more in the discussions. This approach was taken to explore such views at depth and ensure that we had a rich data. We also selected a few other participants who were mostly quiet in order to give them a chance to express their opinions but did not find that they engaged more during the interviews than during the dFGDs. IDI sessions took between 30 and 80 min. Data saturation was discussed by DR and JDV. The dFGDs and IDIs for parents and caregivers were conducted in Setswana while a mixture of Setswana and English was used in the adolescents’ dFGDs. Field notes were made both during and after the dFGDs and IDIs. All discussions were audio-recorded, transcribed and translated into English.

### Participant demographics

This study enrolled a total of 93 participants; 44 adolescents and 49 parents and caregivers attended the initial dFGD meetings. The group size for parents and caregivers’ initial dFGD meetings consisted of about 4–11 participants, while adolescents initial dFGDs had about 5–12 participants. A total of 34 adolescents and 38 parents and caregivers were retained to attend the follow-up dFGD meetings. For these meetings, parents and caregivers’ groups ranged between 4 and 8 participants, and 2–11 for adolescents. The 21 participants (10 adolescents and 11 parents and caregivers) who could not attend the follow-up dFGD meeting had other competing interests. Twelve dFGD meetings with 6 groups of parents and caregivers, and another 12 dFGD meetings with 6 groups of adolescents were conducted, making a total of 24 dFGD meetings. Follow-up IDIs were conducted with 6 adolescents and 6 parents or caregivers (see Table 1 below).

**Table 1:** Summary of dFGDs and IDIs meetings

	dFGD				IDI
	# Groups	# Events	# Participants		# Participants
			Meeting 1	Meeting 2	
Parents/caregivers	6	12	49	38	6
Adolescents	6	12	44	34	6
Total	12	24	93	72	12

Majority of the adolescents (61percent) were female, while 39% were male. Most adolescents (36%) were 16 years old and 93% were either attending junior school or high school or had completed the qualifications. Half of the adolescents lived in villages surrounding the city of Gaborone where this study was based, while 39% lived in the city.

On the other hand, parents and caregivers’ participants were mostly female at 92%, with only 8% of male participants. One reason for this may be that women are usually the primary caregivers in Botswana [38–40]. Most parents and caregivers (39%) were aged between 41 and 50 years and 94% had attained educational qualifications ranging from primary to tertiary education. Similar to the adolescent population, majority of the parents and caregivers (57percent) lived in villages surrounding the city and 43% lived in the city of Gaborone (see Table 2).

**Table 2:** Summary of participants’ demographics

Adolescents	Frequency	Parents and caregivers	Frequency
Male	17 (39%)	Male	4 (8%)
Female	27 (61%)	Female	45 (92%)
Total	44 (100)	Total	49 (100%)
Age		Age	
15 years	8 (18%)	21–30 years	6 (12%)
16 years	16 (36%)	31–40 years	17 (35%)
17 years	11 (25%)	41–50 years	19 (39%)
18 years	7 (16%)	51–60 years	6 (12%)
Unknown	2 (5%)	Above 60 years	1 (2%)
Total	44 (100%)	Total	49 (100%)
Educational level		Educational level	
Primary education	0 (0%)	Primary education	6 (12%)
Junior School	31 (70%)	Junior School	19 (39%)
High School	10 (23%)	High School	12 (25%)
Tertiary	0 (0%)	Tertiary	9 (18%)
None	1 (2%)	None	2 (4%)
Unknown	2 (5%)	Unknown	1 (2%)
Total	44 (100%)	Total	49 (100%)
Residence		Residence	
City/Town	17 (39%)	City/Town	21 (43%)
Village	22 (50%)	Village	28 (57%)
Unknown	5 (11%)	Unknown	0 (0%)
Total	44 (100%)	Total	49 (100%)

### Analysis

All audio recordings of the dFGDs and IDIs were transcribed verbatim and exported into NVivo qualitative data analysis software Version 12 (QSR International Pty Ltd, 2012) for coding and data analysis. The data was coded by two members of the research team (DR and JDV), following an agreed description of the coding framework. Themes were derived both from the literature (deductive approach) and from the data (inductive approach). The qualitative data was analysed using the Framework Method for data analysis [41]. This method encompasses five key stages that are closely aligned with the traditional analytical process of thematic analysis and involves: (a) Familiarisation with the data, (b) establishing a thematic framework, (c) indexing, (d) charting and (e) data interpretation. The Framework method results in the production of a matrix in which cells of summarised data are presented in rows (cases) and columns (codes) ensuring that data can be easily analysed both by theme and by participant or event [42].

## Results

Almost all participants considered that feedback of individual genetic research results could be a way of appreciating participants’ contribution to research. Whilst they gave a range of reasons to support this view, what seemed to lie at the basis of these views were expectations of solidarity and reciprocity.

### Awareness of genetics

As indicated under the methods section, the dFGD method that we used involved an interactive information session to equip participants with a basic knowledge about genetics and genomics research. During the information session, we established participants’ understanding of genetics and found that a couple of them already had a fair knowledge of what genes and genomics are. These participants described genes in relation to family traits, like how people look, how their noses are shaped, intelligence, and diseases that run in the family. After the information session participants were also able to integrate the information they had learnt to make informed responses to the discussions. For example, using a scenario about a mother for whom researchers discovered a genetic predisposition for breast cancer while taking part in a genetic study on mental health, participants were asked about what researchers should do with this extra genetic result. In response one parent said:

My thinking is that, it has been said that a gene doesn’t mean sickness, but I think researchers can investigate and find something that they did not expect. […] So, what I think they could do is to tell her, also taking note of the outcomes, that this is just a gene and not sickness, but it could happen that it could develop into sickness as time goes on. This will help her to prepare herself mentally, knowing that she is living with that gene. And if they tell her when there is still time, she could also take care of herself before the sickness develops. It’s important for her to know before she gets sick, so that she can try to prevent the sickness (P4G5).

In the above quote, the participant was able to clearly articulate that having a gene for a particular disease does not mean one has that sickness and that behaviour impacts on whether or not a genetic predisposition develops into a disease. We found that most of our participants had similar perspectives. Additionally, participants were aware that genes are passed on from both parents and that it is possible for a gene to skip some children and be found in others or skip an entire generation.

### Research participation should be reciprocated

Participants generally reported that they would want to receive their individual genetic research results in exchange for their participation in research. For instance, one adolescent expressed that they "should get something in return if they are taking part in a study" (A1G1). Most adolescents specifically indicated that they would like to get their individual genetic research results back so that they could know how to take care of themselves and behave in ways that would improve their health. Both parents and adolescents often reasoned that researchers should feedback their individual genetic research results because the research would not have happened without them. In particular, they mentioned that they gave blood to be used in the study as well as their time and opinions. One parent noted that:

… They have to tell you what they have found after doing the investigations and you also have to be satisfied, because when doing research, the researcher comes to me to request for my participation…all the questions and answers come from me so I also expect that, after you have investigated on me… that package that you are going to give me should include answers to me, especially what you have found in your investigations including overall findings from participants (P4G5).

A few parents also added that, although research participants usually take up risks that other people would not take, research usually benefits researchers, the university and the nation while participants do not usually benefit directly from research. They viewed feedback of individual genetic research results as a possible way to off-set the potential risks that they could have incurred. They pointed out that receiving individual genetic research results could help them know their genetic make-up. One parent mentioned that:

..it was painful for the children when blood was drawn, it is uncomfortable to be in that position but since we have taken a stand, I think we should be the first ones to get help when we need it because we stepped up […] taking part in research involves everything in you, it is emotional, physical and spiritual. Research should be tit-for-tat, help me, I will help you because we are on a mission that should be accomplished (P4G3).

### Research participation is a mutual relationship that works both ways

While participants were aware that their participation in research could help the nation at large as well as future generations, some adolescents reported that they would like to get their individual genetic research results because otherwise "some people might think that researchers just wanted to know their results only…" (A4G3). This highlights a concern by participants that if researchers do not give participants their individual genetic research results, it would appear like researchers only needed participants to achieve their agenda, without having any concern for their wellbeing. Furthermore, participants expressed viewing research participation as a relationship in which both researchers and the participants are helping each other out. They expressed that since participants helped researchers to advance their agenda by participating in the study, it was therefore appropriate to also expect researchers to advance the knowledge of participants by giving them back their individual research results. One parent expressed that:

Appreciation should be both ways, the researchers have helped me as the participant to find out about genes I didn’t know I had and the participant has also helped the researchers complete their studies by taking part in the research (IDI-P003).

Participants also viewed the research process as a team effort, which meant that they were working together with researchers as a team and helping one another to achieve the goal of promoting good health. One parent expressed that:

…Both the participant and researcher are helping each other out. That we are working as a team. We have to work as a team because we would be working together and agree on issues as you would have helped me with information I didn’t know about (IDI-P003).

### When reciprocity obligations are respected, participants feel valued

Participants additionally expressed a view that receiving their individual genetic research results would show them that their participation was valued. One adolescent reported that "participants want to feel appreciated and motivated to do the right thing” (IDI-A002). A couple of parents also shared this view and added that giving participants their individual genetic research results shows "the participant the results of their participation and that it was valued" (P12G5). Parents also expressed that this would be a learning experience for them but will also show that researchers care about them enough to give them information that could improve the quality of their life. They further pointed out that showing appreciation could encourage participants by knowing that their participation was adding to something and has yielded results. One parent noted:

Yes, it shows the participant the results of their participation and that it was valued. It won’t be right for our blood to be taken for testing and we don’t receive results, because if we were told that they will be testing something then when results are returned, we will know whether we have that thing or not. And this will motivate me, knowing that my participation was beneficial (P12G5).

### Receiving individual genetic research results was valued over financial rewards

Obviously, there are other ways to respect reciprocity obligations in research. Some participants also mentioned other ways of showing appreciation for their participation in genetic research including: giving financial rewards; a gift basket; food; T-shirts; certificates; and a party. We therefore specifically probed whether those kinds of contributions would be an acceptable, alternative to respecting reciprocity obligations.

Both adolescents and parents generally did not support the idea of getting financial rewards beyond compensation for transport and meals and stated that participation in research is voluntary. Most participants indicated that they would rather get their individual genetic research results back so that they can gain knowledge from participating in the study and know how the research is going as well as how their samples are helping. They also mentioned several objections to sharing financial rewards. One adolescent said:

I don’t know about financial rewards because what if it’s not enough money because there are people who are never satisfied by what they receive. I would be so happy to get results because it shows that you guys care, I don’t agree with financial rewards, it’s a no (IDI-A006).

Additionally, participants expressed that such financial rewards may not be a good way to appreciate participants’ contribution as “people might think that their participation was bought" (A4G3). Also, a lot of people may end up wanting to participate in the study only because of the financial reward. They pointed out that research participants "shouldn’t get anything else except their results because they were in the study" (A5G6). Nonetheless, one parent with professional research experience cautioned that even asking to receive individual genetic research results could be problematic for the reasons shared below:

If you now say I want my results then at the end you will say, no…for me to take part in the study, I need 50,000 Pula [referring to Botswana currency], its going there and you will wonder if you can get the best sample or question the quality of the information from such a sample, in most cases it’s a no, and imagine in that case we take prostitutes because they want money, taking people who want to go and drink, so you will not just take anyone, so the statistics principles would already be violated (IDI-P004).

As a result, he pointed out that he would rather have a situation where research is voluntary, and researchers have the discretion to decide to give participants whatever information they deem to be important to share. However, even with this view, this participant also seemed to have held some hope of receiving some form of indirect benefits sometime in the future as a result of having participated in this study:

The research is not necessarily for me as an individual but for the nation so I will benefit in one way or the other. The benefit is in my society that I live in, I have a generation that will keep on moving even when I am gone, that is the generation that will benefit, that is what is important. It’s not me, that is why even today it is said to give generously but by doing so you shouldn’t expect to be given anything back but your reward will come, someone, somewhere will give to me or my generation; my children to what I have given today (IDI-P004).

### Not respecting reciprocity expectations could have consequences

Participants also expressed that there is a general feeling that researchers often need help from participants but would not want to help them when they need help. However, they expressed that failing to reciprocate to participants by researchers could have some negative consequences as it will reveal that researchers do not want to help participants after participants helped them to achieve their agenda. When asked about who they thought was responsible for covering costs of follow-up care and why, one parent said:

I think it’s because they feel that you need their help but wouldn’t want to help them when they need you, that is why they expect you to help in getting other tests because you would have found what you were looking for so at least help with subsequent tests, that help you offer the participants could encourage others to keep volunteering for other researches as this isn’t the only research that will be conducted, there could come another study that needs participants, I could discourage others from taking part in studies because of the way I was handled during my time when I was participating in this research and if they hear me say that, then they might not take part, or I could tell them that I benefited from taking part in a study and people would be motivated to take part in more research studies, that is why they expect that sort of assistance from the researchers, so that they don’t lose motivation and keep advising others to take part in researches (IDI-P006).

## Discussions

In this study, we sought to understand how solidarity and reciprocity impact on African researchers’ obligations to feedback individual genetic research results by examining views of adolescents and parents in an HIV-TB genomic study in Botswana. We found out that almost all participants considered that feedback of individual genetic research results would be a way of appreciating participants’ contribution to research. Whilst they gave a range of reasons to support this view, what seemed to lie at the basis of these views were expectations of solidarity and reciprocity. Participants viewed research participation as a mutual relationship that needs to be respected. They also saw themselves as working together with researchers as a team, helping one another to achieve the ultimate goal of promoting good health*.* The principle of solidarity calls for team members to look out for each other and act in ways that could benefit one another. As Metz [43] indicated, “*once a researcher and a participant have begun to think of themselves as a ‘we’ engaged in the joint project of a study, they have formed a tie that imposes special obligations to care for one another’s quality of life that can go beyond those listed in a participant agreement form (with the one in a greater position to aid naturally having more of a duty to do so)*” (p. 117.) As result since participants helped researchers advance their knowledge, it would be appropriate for them to also help participants advance their knowledge by giving them their individual genetic research results. This finding resonates with the ancillary care model developed by Richardson and Cho [11] who highlighted that research participation means that researchers enter into a relationship with participants, in which they take on limited responsibility for the health of research participants. The nature of this relationship is such that they have ancillary care obligations for findings that are relevant to the health of the condition under study. However, the ancillary care model advanced by Richardson is slightly different from the solidarity and reciprocity argument advanced in this paper, as pointed out by Metz [43] the nature of the relationship advanced in Richardson’s ancillary care model is a result of research participants’ having entrusted researchers with their bodies and information, therefore it is this “reduced privacy or compromised autonomy” (p.121) that obligates researchers to provide support to participants. As a result, this limits the range of support that a researcher could provide to participants as it could exclude other conditions that participants may have, even if they were known to the researcher prior to the study [43]. On the other hand, while solidarity and reciprocity argument advanced in this study are also relational, the difference is that the relationship here is communal. According to Metz [43], “*upon sharing a way of life with participants, a researcher has established part of a morally significant relationship that demands respect and hence full-blown realization in the form of caring for their quality of life as well*” (p. 117). Unlike the entrustment model where the duty to provide help is a result of the disclosure of private information, this appeal to communion could possibly have a much broader reach [43]. In our study, participants were less worried about ancillary care obligations but framed that the nature of reciprocity obligations required that researchers return the value of their research participation *in kind.* Importantly, what that means to them is that where their research participation helped researchers improve knowledge, so too should researchers help them improve their knowledge.

In addition, participants noted that providing them with their individual genetic research results could off-set the risks that they would have taken up by participating in the study. According to Prainsack [26], a solidarity-based viewpoint means that we can recognize that those who volunteer to help others, often take-up some costs, and that even though such acts are often viewed as gifts that are voluntarily given [44], the nature of the gift is influenced by social relations and shared responsibilities that exist between the giver and the recipient [26]. Therefore, once the gift is given, it often creates a “web of indebtedness and future reciprocity” [45]. Our participants’ balancing the risk that they took on with their views on return of results could perhaps be understood in this light.

Our findings further revealed that, when reciprocity obligations are respected, participants feel appreciated and valued. This in turn enhances solidarity between participants and researchers and could motivate participants to participate in future research, knowing that their participation was beneficial and has yielded results. Participants highlighted that they need to feel that they are part of something larger and that their contribution fed into a reciprocal, respectful and beneficial relationship. Honouring reciprocity obligations is important in maintaining societal relationships and stability.

While there are many ways to reciprocate participants, including financial incentives, many participants in our study did not consider monetary rewards beyond compensation for transport and meals; as an appropriate way to reciprocate. They mentioned that such financial incentives could undermine the voluntary nature of the contribution. This is particularly important, as it means that participants appreciate that research participation is a voluntary endeavour and that getting money for research participation may make it seem like a commercial exchange rather than a relationship based on solidarity and reciprocity, where any exchange of gifts is based on caring for one another. Prainsack [26], states that in a solidarity model, what the gift giver should be able to anticipate from the one who receives it, is that in reciprocating, the recipient will treat her as per the same standards that have been accorded to her. As a result, a solidarity outlook requires that we work toward attaining balance and reciprocity between those who are within the organisational and institutional context in which the “gift” is given. This suggests that it makes sense to expect researchers to compensate the knowledge-based benefit that participants have accorded them by providing a knowledge-based benefit to participants. Returning overall study results is another way of achieving that, but participants in our study specifically expressed that they would want to receive individual genetic research results as a form of appreciation for their contribution. These views are consistent with findings by Marsh et al. [36] in Kenya, who found that disclosing individual genetic findings about sickle cell disease was strongly supported by participants due to perceived health and social benefits. Several other studies conducted elsewhere also revealed that participants generally would like to be provided with feedback on their individual genetic results [46–51].

## Conclusions

Overall, our findings seem to suggest that our Batswana research participants conceptualise participation in genomics research in terms of a reciprocal relationship. They articulated an expectation that their gift of research participation, which helped researchers produce knowledge, should be reciprocated by sharing knowledge-based benefit—as would be provided when sharing individual genetic research results. They were therefore overwhelmingly in support of an obligation for researchers to share individual genetic research results. As a result, these expectations of solidarity and reciprocity by participants could mean that African researchers do have an obligation to return selected individual genetic research results. However, we could not exclude that there are other reasons for the high-level of support for feedback of results.

Obviously, in this paper we did interrogate other, broader aspects relating to the feasibility of returning individual genetic research results—and so, even if in this paper we have established that there seems to be an ethical obligation to return individual genetic research results, we cannot comment on the extent to which this is practicable or feasible.

## Limitations

Although these findings are an important addition to the literature on feedback of results in genomics research in general, our study has several important limitations. Firstly, most of our participants (92%) were female, possibly because women are usually the primary caregivers in Botswana [38–40] due to sociocultural and legal factors [52]. We were unable to attract a large number of males to participate in our interviews even though we particularly encouraged male participation. In doing so, we may have been hindered by the fact that we recruited participants through the existing CAfGEN database which mostly holds contact details of female parents and caregivers. Going forward, more active recruitment directly targeting male participants is necessary to access their views. Secondly, since there was no metric for participant selected for IDIs, it is possible that we may have biased selection of participants in the interview component of our study towards more extravert participants. We sought to remedy this bias by deliberately selecting a few other participants who were mostly quiet but cannot exclude a potential bias in our data towards more outspoken individuals. Thirdly, in our study the feedback of individual genetic research results was presented as a hypothetical situation and the genomic study that we linked our study on does not have an existing plan to return individual genetic research results. It is possible that participants’ views may change when faced with a real situation and this is worthy of investigation once African genomics projects start to implement return policies.

## Supplementary information

**Additional file 1.** Manual for the deliberative focus group discussions.

**Additional file 2.** Guide for indepth interviews.

## Data Availability

The datasets used and/or analysed during the current study are available from the corresponding author on reasonable request.

## Abbreviations

AIDS: Acquired immunodeficiency syndrome
BBCCCE: Botswana-Baylor Children’s Clinical Centre of Excellence
CAfGEN: Collaborative African Genomics Network
dFGDs: Deliberative focus group discussions
H3Africa: Human Heredity and Health for Africa
HICs: High-income countries
HIV: Human immunodeficiency virus
IDIs: In-depth interviews
IFGENERA: Individual Findings in Genetics Research in Africa
IRB: Institutional Review Board
LMICs: Low and middle-income countries
TB: Tuberculosis
