# Supplementary material for: Do solidarity and reciprocity obligations compel African researchers to feedback individual genetic results in genomics research?
Source: BMC Med Ethics. 2020 Nov 4;21:112. doi: 10.1186/s12910-020-00549-4 (PMC7640670; doi:10.1186/s12910-020-00549-4)
Supplement: Supplementary file 1 — Additional file 1. Manual for the deliberative focus group discussions. [file 12910_2020_549_MOESM1_ESM.pdf]

**Manual for Deliberative Focus Group Discussions (dFGDs) on Feedback of Individual Findings in Genomic Research among Lay Individuals who have Participated in Genomics Research in Gaborone, Botswana, and Cape Town, South Africa**

*Developed by Elsie Breet and members of IFGENERA project 2, along with guidance from Vicki Marsh, between April 2018 and March 2019.*

***Important note:*** *This manual may not be published in any form. Sections of this Manual have been guided by the 'Manual for Community Consultation Information session on Benefits and Payments to Research Participants in Kilifi' by Vicki Marsh and colleagues. The manual may be used for the design of other dFGDs on topics similar to the one explored in the IFGENERA project.*

**Version 1: January 2019**

| TABLE OF CONTENTS | | Page |
|---|---|---|
| **1.** | **Research study outline** | 3 |
| 1.1 | The background and rational | 3 |
| 1.2 | The overall aim of the research | 3 |
| 1.3 | How will the research be undertaken | 3 |
| 1.3.1 | Encourage differing opinions | 3 |
| 1.3.2 | Participants | 4 |
| 1.3.3 | Data collection procedure | 4 |
| 1.3.3.1 | Initial information session | 5 |
| 1.3.3.2 | First round of questioning | 5 |
| 1.3.3.3 | Data analysis of dFGD 1 | 6 |
| 1.3.3.4 | Second round of questioning | 6 |
| 1.4 | General outline of the information session guide and dFGDs tool | 6 |
| 1.5 | General guidelines for facilitating group discussions and sharing information | 7 |
| **2.** | **Manual for information session and focus group discussion** | 8 |
| | Module 1: Starting the information session | 8 |
| | Module 2: Basic understanding of genetics and heredity | 10 |
| | Module 3: First deliberative focus group discussion | 16 |
| | Module 4: Closing the information session and next steps | 22 |
| | Module 5: Second deliberative focus group discussion | 23 |

## 1. RESEARCH STUDY OUTLINE

### 1.1. The background and rational

The decision to feedback individual genetic findings is a complex issue. Researchers may be steered by the desire to respect research participants by feeding back relevant individual findings in genomics research. However, the final decision could be challenged by the responsibility to protect participants from the level of uncertainty associated with research findings that have not been validated for diagnostic purposes. Traditionally, researchers have opted to err on the side of caution and not to feedback too much of what has been found. The result is a phenomenon described as 'helicopter research' where research teams move into the lives of individuals or communities to collect personal information and then leave without any sort of feedback or communication thereafter.

Some research suggests that individuals carry varying levels of curiosity about themselves, particularly when it comes to their own health and wellbeing or that of their family. As such, participants might experience feelings of loss and frustration when research studies take information from them without sharing what was found. This is not only damaging to the work done at the time but could also hinder any future attempt for research or interventions in these same communities. In struggling with these competing and contrasting ideas, the objective of this research is to understand what African research participants think about disclosure of individual findings in genomics research. This knowledge holds important implications for engagement, collaboration, and transparency between research participants and researchers.

One important question in discussions around the feedback of individual genetic findings generated in the context of genomics research, relates to stakeholder expectations of and preferences for which findings are to be fed back, to and by whom, and when. This study will investigate key questions related to what people may want to know, why they want to know it, and how they would like to receive such results. Other questions relate to reciprocity / solidarity, whether research results need to be verified in a diagnostic laboratory, and how important it is that participants who receive results, have access to the interventions that would mediate the effects of the genetic finding.

### 1.2. The overall aim of the research

The specific aim of this study is to explore expectations and preferences for feedback of individual genetic research findings with (parents of) participants involved in genomics research in Botswana and South Africa.

### 1.3. How will the research be undertaken?

- **Encourage differing opinions**

We will achieve the aim of this study by conducting deliberative focus group discussions (dFGDs) with a diverse set of research participants in genomics research. Diversity among participants is important to ensure the collection of competing ideas that can challenge our current understanding of the issue, with the objective of generating a rich description of the range of perspectives on this topic. We can introduce diversity into the group by recruiting individuals who vary according to age, gender, level of education, experience of engaging with research, religion or culture.

- **Participants**

Specifically, dFGDs will be used to explore preferences for the extent, nature, timing and means of feedback of individual genetic research findings in African genomics research. This component of our study will take place in Gaborone, Botswana and Cape Town, South Africa, using three ongoing genomics research projects:

- In Botswana, we will involve parents of children and adolescents involved in the H3Africa CafGen project;
- In South Africa, we will involve parents of children and adolescents involved in genomics research in the NeuroDEV (i.e. children with neurodevelopmental disorders) and the NeuroGAP (i.e. adults with psychotic disorders predominantly schizophrenia and bi-polar disorder) studies.

We will conduct 27 dFGDs with the a) parents of children, b) adolescents, and c) adult participants enrolled in genomics research in Botswana and South Africa. We will categorize findings that could potentially be fed back to research participants in categories varying in degree of actionability and predictability and discuss with participants whether and why they may want to receive those findings.

- **Data collection procedure**

We will engage each participant in three separate activities (as groups) namely 1) an information session and 2) two small group discussions (deliberative Focus Group Discussions or dFGDs) held over two days (see Figure 1).

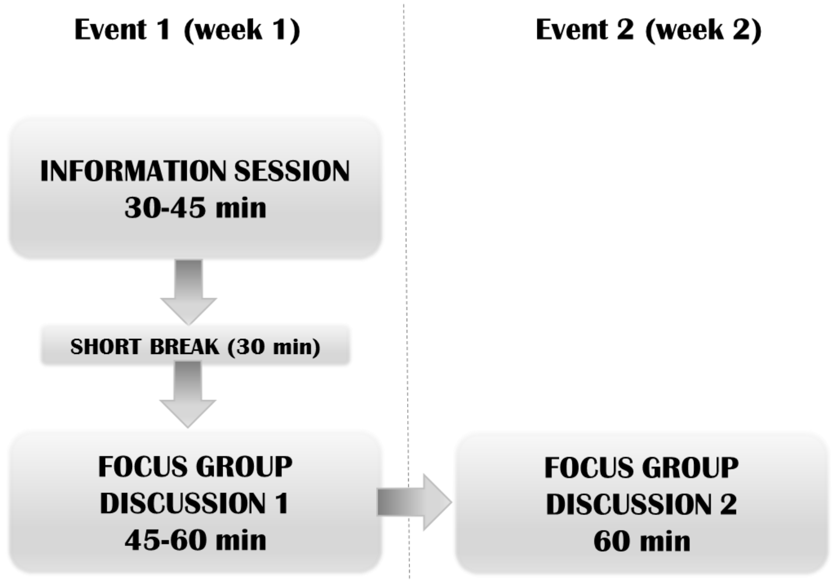


*Figure 1 Overview of research process*

During the information session we will discuss genetics and heredity, how genetics may inform on health and what participants may expect from the research. During the information session, we will pay careful attention to how individuals make sense of genomics research and genes. This is important to gauge whether individuals are comfortable in discussing the material in the dFGD or if further information is necessary to prepare participants for the dFGDs. During the small group discussions participants will engage in discussions about what findings should be returned, when and why. The first small group

discussion will take place on the same day as the initial information session. The second small group discussion will take place within two weeks of small group discussion 1. The reason for planning the dFGDs in two stages is: 1) to foster reflection and to allow enough time for information sharing on the topics discussed during the information session as well as the first round of dFGDs, and 2) for participants to go home and think about these issues, perhaps to talk to others, before returning for the second dFGDs.

- **Initial information session:**

We aim to conduct a short 30-45 minute initial information session. We will use this information session as a learning tool for participants and for the research team. This will also be an important opportunity to assess whether our planned research process is appropriate. We will hold information sessions at a venue convenient to participants – in Cape Town, they will be facilitated at the hospitals where NeuroDEV and NeuroGAP recruitment is taking place whilst in Botswana the research team may decide where the sessions are best held. During the information session we will take notes of the discussions, but these will not be audio-recorded.

The first part of the information session will be spent talking about genetics and heredity. During this part of the session we will: create a space where people can discuss that genes are things that are carried with us in our blood; talk about family inheritance; and weave in ideas that heredity is about chance / God's Will. The second part of the information session will be spent on explaining: that genomic research is predictive of something but it is not an absolute; that genomics research is not diagnostic; what findings can be expected from research; and what can be expected from the research process. Once we feel comfortable that participants have a fair understanding of what genomic research is about, we will complicate the narrative by explaining that sometimes research can find something about the health of a participant and providing some information about what genomics could reveal about a person's health.

***Look out for:*** *During the information session we will make use of case studies to facilitate the discussion. These case studies will use indicator illnesses like breast cancer as an example of what can be found out about the health of a participant following their participation in genomics research. However, it is important to note that* <u>we are not primarily interested in knowing a lot about the conditions</u> *in the case studies (i.e. we're not fostering discussions about breast cancer) but that the indicator illnesses are used* <u>simply to facilitate</u> *a discussion about return of individual results in genomics research.*

- **First round of questioning:**

For the first round of questions we will conduct group discussions with groups of participants who have provided written informed consent that they would like to participate in the dFGDs and have completed the information session. The number of small groups enrolled for the dFGDs will depend on the number of people who participated in the initial information session. Each dFGD will consist of a maximum of 6 participants to give each participant an opportunity to partake in the discussion. The discussion will be recorded with permission.

- **Data analysis of dFGD 1:**

After dFGD 1 the interviews needs to be transcribed and analysed before the dFGD 2 meeting. This is important so that the interviewer can make notes of key relevant issues that need more clarity or can be explored / expanded on during the dFGD 2. If it is not possible for the interviews to be transcribed and studied carefully before the dFGD 2, then the interviewee needs to listen through the recording of dFGD 1 and make notes of key issues and areas that need more clarity or can be explored / expanded on during the dFGD 2.

- **Second round of questioning:**

Within 2 weeks of the information session and dFGD 1, all participants will be invited to attend a second round of questioning (dFGD 2) to further explore and reflect on views discussed in the information session and the first round of questioning. The discussion will be recorded with permission.

  - ***Phase 1:*** Phase 1 of the second round of questioning will be used to present case study 1 again in order to recap some of the answers participants provided during the first round of questioning. The aim of the recall is to challenge participants about their views and opinions raised during the first round of questioning. The interviewer can use this space to introduce more complex questions and counter questions as possible probes that might enrich participants' answers during the first round of questions.
  - ***Phase 2:*** Phase 2 of this session will be used to discuss relevant information that has come up during the data analysis of dFGD 1.

### 1.4. General outline of the information session guide and dFGDs tool

The information session will provide information about basic aspects of genomics research to ensure that participants have enough awareness of some of the key issues to sustain an informed discussion about feedback of individual findings during the dFGDs. Information sharing should be done in a way that participants feel comfortable and empowered to engage with sometimes complex issues in genomics research.

The information session guide comprises of different modules that work together to allow a combination of information sharing and exploration. The two modules that make up the information session are::

  - **Module 1** provides an introduction and a rationale for why the research study will be undertaken.
  - **Module 2** establishes some basic information about genomics research and what it can reveal about health. The module includes suggested questions that could be helpful to facilitate the discussion and check understanding among participants.

The small group discussion tool is a semi structured guide that will be used by the facilitator in navigating through the focus group discussions. The guide is carefully structured in a way that will allow a detailed probe into the views and reasoning of participants. There will also be an opportunity for other participants to share their opinions on the views raised by other participants.

Some instructions for the facilitator have been included in italics to highlight things to look out for or follow up on during the discussion.

The small group discussion tool consists of three modules:

- **Module 3:** presents questions and prompts for the first round of dFGD which is facilitated by the help of a case study scenario.
- **Module 4:** provides details on closing the information session, dFGD 1 and planning next steps for dFGD 2.
- **Module 5:** presents questions and prompts for the second round of dFGD to explore in more detail and challenge the views and ideas raised by participants during dFGD 1. Some additional questions to further complicate the session is also included. This session is facilitated with the help of notes made during the information session and preliminary analysis of the discussion for dFGD 1.

### 1.5. General guidelines for facilitating group discussions and sharing information

- Note that this discussion is not about getting participants to agree on the topics that are being discussed, instead we need to try and develop a comprehensive understanding of all views, concerns and opportunities around the feedback of individual genetic research results in African genomics. It is therefore essential to try and explore different issues while carefully considering the reasoning behind these issues;
- In order to understand how participants think about the views or opinions they share, it is important to probe why particular people hold certain views, to offer counterpoints and to work together with participants to interrogate assumptions;
- It is necessary to keep a watchful eye out for participants who dominate the discussion. The facilitator needs to remain in control of the group and give all participants an opportunity to share their views. This means that it will sometimes be necessary to direct questions at those participants who have not contributed to the discussion. Some caution is necessary when doing so in way that encourages view sharing but avoids discomfort among the participants;
- Take care not to lead participants during the discussion, we need participants to share their own views. Equally important is to not show reaction (i.e. positive or negative) to views shared by the participants, instead remain neutral and non-judgemental so that participants can feel comfortable to share their own views;
- It is important to take notes during the discussion. Carefully note the ways in which participants say things and try to write down any unique phrases or words. These notes can then be used to further guide the discussion or probe in more detail about relevant topics;
- Ideally each discussion group should have a co-facilitator. This is helpful because the co-facilitator may be able to ask questions and ask for clarification which the facilitator might have missed.

## 2. MANUAL FOR INFORMATION SESSION AND FOCUS GROUP DISCUSSIONS

### MODULE 1: Starting the information session

**Background to the module:**

- In this module it is important to create an open, friendly and safe environment where participants feel free to participate in the discussions. This is important since we want participants to feel comfortable to share any opinions or views in an environment that is free from judgement or other negative factors that might limit participation.
- The data collected from notes and recording of the dFGDs, will primarily be used as the data for two PhD degrees, reports, and peer-reviewed published papers.
- It is essential that we obtained signed informed consent before the start of Module 2. This process will entail carefully reading through the informed consent, along with participants, and asking each participant to indicate their willingness to participate in the study by signing a paper-based version of the informed consent form. We will also provide a copy of the informed consent form for participants to take home and keep for their own record, with contact details for the primary investigator and ethics committee in case they have any concerns or further questions.

**Outputs of this module:** When this module has been completed, the facilitator: must have established an open, friendly and safe environment where participants feel free to participate in the discussions; must be sure that participants understand what will be expected from them and how this process will take place; and should feel confident that all participants are comfortable with the way in which the data will be used (e.g. obtaining degrees etc.). Each participant will also have had to sign an informed consent form.

**The sections below should be explained in your own words but make sure to consistently check understanding (e.g. using open questions) among participants.**

**1. Welcome/Introductions:**

- Welcome the participants to the dFGD and express your appreciation that they are willing to take part in the discussions. Facilitator(s) also need to make sure to introduce themselves;
- State the general aim of the information session as understanding how people think about genes, family inheritance, and genomics research as well as what is expected from our research to make sure that research is done in a way that is fair and safe;

**2. Objectives of the information session and of the research:**

- The information session is an important part of a process that will produce data that will form part of two separate PhD degrees and inform practice in the way the group conducts genomics research.

**3. What to expect**

- This information session should be used as an opportunity to tell participants about the research process. We will also conduct the first dFGD after the information session;
- All participants will be invited to attend a second dFGD within the 2 weeks following the information session and dFGD 1 to continue these discussions. During the information session, participants should also be given the names of participants to attend the various group discussions;

- There will be a reimbursement for time and travel costs (Give amount for SA and Botswana) for each session.

**4. Informed consent process**

Read through the informed consent carefully and make sure that every participant indicates they understand what is expected of them. Each participant should sign a copy of the informed consent form. The points below should be emphasised before going on to the next step of the informed consent form.

- **Remind the participant of study participation**

Your participation in this study is voluntary. If you agree to participate in this study, you will be asked to participate in a group discussion. The total time we will use today is about three hours. You will be asked questions about genomics and individual results. If the questions make you uncomfortable, you can tell us and you don’t have to answer. You may withdraw your participation from the discussion at any point, even after you have initially agreed to participate in the study.

- **Remind the participant on confidentiality statement**

To help us with our research, we would like to record the conversation. We will write down the conversation, but we will not write down your name and any other information that can identify you. Therefore, no one will be able to identify you from the transcript. As a participant in this research study, you will be assigned a number. Therefore, no one will be able to identify you from the database. Any publications or other research outputs based on this data will not identify you directly. The data will only be accessible by the research staff working directly on this study. The research staff are researchers and PhD students working directly on this project.

- **Recordings or photographs**

We would like to ask for your permission to record the information session as needed and to take some photographs. If you provide your permission at the start but change your mind during the session you may ask us for any information you would like us to exclude.  you are still aloud to change your mind at a later stage should you want to.

- Give participants an opportunity to ask any questions or raise issues about the recordings.
- Ask for written informed consent from each participant.

**5. Housekeeping issues, including:**

- Make sure that all participants know of any breaks (e.g. tea) and when the session will be completed;
- Provide clear instructions of where the bathrooms are;
- Address transport refund issues.

**MODULE 2: Basic understanding of genetics and heredity**

**Background**

- This module has the main aim of drawing out what participants understand about genes and heredity. We aim to listen to the way that participants describe the research components from what is necessary to understand our research. This is an important step in the information session as participants will not be able to comment on the how, what, when, why or why not of feedback on individual research findings if they do not have a basic understanding of what these findings relate to.
- Because genomics research is a complex issue that will be difficult to talk about among lay individuals (*also influenced by culture, language, personal life experiences*) in general, the discussions will focus on key concepts needed for a minimum understanding to participate in the dFGDs. Concepts will be explained with the help of a story that is more relatable for participants. Even though the story has been made to be simple, the content may be new and difficult for people to grasp immediately. To help understanding, the story box should be explained as a narrative on what happens to a particular named individual and with enough details of the family for participants to begin to build a mental picture of this situation. The facilitator can use the story to illustrate basic concepts in genomics research. Facilitators should probe comprehension by asking open questions and repeating information where needed. These reminders may continue to be needed throughout the discussion of the sessions, and the facilitator should be on the look-out for any signs that views are being expressed because participants have misunderstood or forgotten important aspects of the example story.
- The concepts discussed will also be used as the focus for discussions in the dFGDs, and one aim of introducing them in the information session is to help people to quickly pick up on these issues in these discussions.

**Outputs from this session:** By the end of the module, you should be fairly certain that people grasp the key points in genomics necessary to facilitate a discussion about the return of results.

**Follow the instructions and use participatory methods to explore the issues described in the text below along with any other relevant issues that may come up.**

1. **Genes and family inheritance**

- ***Introducing story 1: Genes and how they are passed on in a family***

In the next set of discussions, we would like to find out what you know and understand about our research and how we find out the things that we do. To understand this, you need to know a little bit about genes and how they are passed on in families. We know that you might not have heard of these things before but don't worry, we are going to talk about this now until every person knows what these things are.

To help us talk about these topics, we will give you some examples and explain these to you as a story, and we will ask you to talk about what you understand from what was said and what questions you might have. So, while you are listening to the story, please try to pick out anything that stands out to you.

***For the facilitator:*** *At the end, check understanding of the main points in this scenario.*

**Story 1[1]: Fezile meets the family and learns about genes and family inheritance**. It is finally the day of the wedding. Fezile holds his mother's hand tightly as he feels excited to attend his first ever family wedding, but he is also a little nervous about all the mostly familiar faces starring back at him. Some of his aunts, uncles, and cousins he has not seen since he was younger come over to great them excitedly.

Aunt Noluthando pulls Fezile into a hug and says, "Look how you've grown! And you've got such a beautiful smile and big bright eyes! You remind me so much of my grandfather!"

Uncle Lwazi adds, "Look!, He is getting so tall and strong! You look just like your father. Only 11 years old, and you look like a soccer player! It runs in your blood!"

The wedding is beautiful, and the guests have lots of fun celebrating after the wedding ceremony. That night Fezile has trouble sleeping and wonders, "Aunt Noluthando's grandfather? It runs in your blood? What were they trying to say?"

The next day Fezile tells his mother what happened and asks her to explain what his family members were talking about. His mom, who is a science teacher at the local school, excitedly tells Fezile: "Genes! that is what they are talking about!"

➢ ***Discussion***

In a large group, use the topics highlighted throughout this section to discuss concepts around genes and heredity by using the information described in the rest of this section. In discussing each of the concepts below, the facilitator should use participatory group discussion methods to ensure that participants are comfortable with these topics. Participants do not need to understand the detail of genetics research and how genes work, only transfer a basic understanding of these points using the description that follows (*see descriptions below)*:

- What are genes?
- Where are our genes?
- Genes are passed on through families from parents to children;
- Genes that are passed on to children are from both parents – we inherit genes from both parents;
- Genes play a role in physical appearance e.g. the shape of our face and height but also other characteristics **AND** functions inside of the body that cannot be seen with the eye e.g. mechanism that controls breathing;
- Heredity is about chance (which participants may know or understand better as 'God's will'). This means that even if you carry a genetic factor, it doesn't mean you will actually develop the disease;
- Sometimes things go wrong in our genes – called mutations – that can make people sick;
- Mutations can be passed on from parents to their children through genes;

[1] Adapted from: Narcisa, L. (2014*). Kids Health: What is a gene?* Retrieved from https://kidshealth.org/en/kids/what-is-gene.html

- Researchers study genes to understand how they work and to find out what mutations make people sick;
- To study genes they ask permission to test blood from people who are willing to take part in their studies.

**What is a gene and where are they?**

*Starting questions:*

- *Who in the group knows what genes or DNA are?*
- *What do genes do?*

  *PROMPT: explore participants' basic understanding of genetics and inheritance*

It can be fun to explore all of the ways that you are like others in your family – for example your height, the shape of your face, or the same talent for a sport like soccer. But why are we like others in our families? Well, because of our genes.

This is not the same thing as the 'jeans' that you buy at the shop. Genes are much more exciting than that. Genes are the recipe for making you the way that you are and they come from your mother and her family and from your father and his family. Your body is made up of cells. Every cell has about 25,000 to 35,000 genes inside of it. These genes hold the information that makes up the features or characteristics that are passed on to you (i.e. inherited) from your parents. The genes are made up of a material we call DNA – you may know that from CSI or other television shows. 'DNA' sort of means the same thing as 'Genes'.

Let us look at some examples. Say both of your parents have funny ears, you might also have funny ears if you inherit this feature from them. Similarly, if your father is very short, there is a chance that you could also be very short because you inherited the feature shortness. Genes do not only belong to human beings; we also find genes in animals and in plants.

You are probably wondering where in our bodies we can find these important genes? Well, genes are very small so we are not able to see them with the human eye. A cell is so small that we have to use a strong microscope to see it. Cells are everywhere in your body and there are billions of them. There are cells in blood, but also in your skin and muscles. Because there are cells in blood, and all cells contain genetic material, researchers can take a blood sample to get some of your genes out of your body.

*Questions to group to probe comprehension:*

- *Do you feel you have a better understanding of what genes are now?*
- *Can you tell me what genes do?*
- *Where can we find genes (or DNA) in the body?*

**How do genes work:**

Every gene is responsible for giving out specific instructions, sort of like a recipe handed down from your mother or grandmother, that tell our bodies to grow, work properly, and stay healthy.

Your parents each have two copies of each of their genes. Only one copy of their genes are then passed on to you so that you have one copy from your father and another from your mother. The pair of genes that you inherit from your parents are responsible for making you look like your parents and your family by making you have their type of body, face and so forth.

In the story that we told you earlier, Uncle Lwasi told Fezile that he is getting tall and strong just like his father. This means that Fezile's father is a tall and muscular man and that he has passed on some of what he looks like to his son.

Just because genes are passed on from your parents it does not mean that you will look and be exactly like them. For example, if both of your parents are tall you might be short. This is because we get different genes randomly passed down over generations – your grandparents might all be short, and even though your parents were tall, you got the genes for shortness from your grandparents. Remember in the story Aunt Noluthando told Fezile that he looks just like her grandfather.

Genes do not only make up how we look. Genes also work inside of our bodies on things we cannot see but that we need to be healthy. For example, we have special genes that help us to carry oxygen in our blood which we need to help our cells function well. We have genes that influence the shape of our heart or how strong our muscles are.

*Question to the group: is this clear to you? Are there any questions?*

**When there are problems with genes:**

So genes tell our bodies how to grow, work well and remain healthy. But sometimes, the genes that we have can also make us sick or weak, so that we can develop diseases.

Sometimes the genes from a mother and a father can give problems to the child. This happens when genes are changed in some way so that they do not work properly. Genes that have been changed are called mutations. This can lead to diseases.

Some of these gene problems can be passed on from a parent. For example, take a gene that can make the heart weak. If that gene is passed on from a parent to a child, the child's heart can also be weak.

Some illnesses that seem to run in families are due to genetic mutations, but these still won't necessarily pass from parent to child in very straightforward ways (e.g. skipped generations – people likely to recognise this in everyday life – as for height, skin colour). Some illnesses are influenced by genetic mutations but also by other things, such as the kind of food you eat, or other illnesses that you have during your life. Some are not due to genetic mutations at all, but other things that might affect families over generations, like living near to a source of pollution, or always having eaten certain types of unhealthy food.

*Question to probe comprehension:*

- *Do you know of any sicknesses that run in families?*
  *PROMPT: heart disease, cancers, psychiatric conditions, other?*

***Heredity is about chance:*** But this does not mean that if your one parent has a mutation that you will also get the mutated gene. Remember that we said that just because your parents are tall does not mean that you will be tall? There will be characteristics and features that you get from your current and past family. Sometimes, a disease that a grandparent or aunt or uncle had can also affect a grandchild, even though the parent was healthy.

***For South Africa NeuroDEV dFGD - De novo mutations:*** but we don't always inherit illnesses from our parents. It is also possible that a fault slips into the DNA when the child is still in the mother's tummy,

before she even knows that she is pregnant. Sometimes it means that the baby is born with a disease that nobody in the family has.

**How can my genes impact on my health?**

As we said before, our genetic information tells our bodies how to work. But sometimes, our genes could also cause our bodies to be weak for a particular disease. We know this because sometimes people live in the same house and eat the same food and have the same behaviour, but one person gets ill and the other doesn't. For instance, you can get two people who both smoke a lot of cigarettes. But one may develop lung cancer and the other doesn't. We think that this is because of the exact shape of the genes they have – for the one person, they may protect him from the lung cancer whilst for the other they may have made his body weaker.

But most times, the exact shape of the genes is not enough to give you an illness. You need to have the genes, but you also need to have a certain behaviour that can cause the illness. For instance, if you have genes that mean that you are at risk of developing heart disease, then that does not mean you will get heart disease. If you exercise, walk everywhere, and eat healthy food, then you can stay healthy. But if you eat unhealthy, don't move AND have genes that mean you can develop heart disease, then you could be at risk. In a way, this is about God's will: even if you carry in your genes a susceptibility to developing a particular disease, this doesn't always mean that you will get it. Research that look at genes could help to identify if we are at risk of developing a particular diseases that can make us sick, but again, research cannot confirm that we will definitely get sick if we have a risk of getting a certain disease.

*Questions to probe with the group:*

- *Does having a gene for a particular disease always mean you get the disease?*
- *What does it mean for something to be 'God's will'?*
- *If a doctor were to tell you that you have a gene for this illness, what does that mean for your health?*

  *PROMPT: use example conditions such as heart disease, cancers*

**Research and why we are having this discussion**

Researchers and scientists are working hard to try and find out how genes work in our bodies. If researchers can find out how genes do what they do, they can know what diseases are caused by genes that don't work properly.

In our research we are also trying to understand how some diseases work and which genes might be the reason for different diseases. To do this we need people like you to participate in our research studies. For the study researchers draw some blood from different people who give their permission to use their blood in research. Genes are carried in cells in the blood so by testing the blood we can find out more about genes and the genes that might play a role in why people get sick. It is very important for us to do research about genes and which problems in genes – mutation – might make people sick because if we know this then we can start making plans of how to help people or how to keep people healthy.

Part of what we want to discuss with you today is what do you think about research where we test genes to look for any problems (mutations) so that we can help people to get better.

It is important for you to understand that the purpose of research is to learn about diseases in a way that benefits all people, not just individuals. When we spend time looking at results that could benefit individual people, that means we can spend less time learning about disease in a way that benefits all people.

*Questions to the group:*

- *What is the purpose of research?*
  *PROMPT: to benefit all people, not just individuals*

- **Summary of module**

Conclude this module by summarising the main points about genes, heredity, and willingness to participate in research. By the end of this session participants should be comfortable with the idea of genes, that genes are passed on through families by chance and that research studies are done among lay individuals like themselves to explore genes and to identify problems in genes (mutations). A basic understanding of these concepts is important before going on to the next section where we will discuss in more detail how participants think about what is found in research studies and how, when, or why these findings should be communicated back to participants.

**<u>Short coffee break with some refreshments: 30 minutes</u>**

## MODULE 3: FIRST DELIBERATIVE FOCUS GROUP DISCUSSION

**Background to module:**

- This module has the main aim of drawing out what participants understand about the return of research results, and what research does or does not do without being unduly influenced by researchers' ideas about these (e.g. 'many researchers believe that results which have limited value to participants should not be returned because this would entail a burden of cost or complexity to return'). We aim to listen to the way that participants describe findings we derive from our research. This is an important step in the information session as participants will not be able to comment on the how, what, when, why or why not of individual research findings if they do not understand what these findings are.
- Because genomics research is a complex issue that will be difficult to talk about among lay individuals (*influenced by culture, language, personal life experiences*) in general, the discussions will focus on key concepts needed for a minimum understanding to participate in the dFGDs. Each concept will be explained with the help of Case Study Scenarios that are more relatable for participants. These scenarios may be difficult for people to grasp at first. To help understanding, the Case Study Scenario boxes should be explained as a story i.e. a narrative on what happens to a particular named individual living in a particular named place and with enough details of the family for participants to begin to build a mental picture of this situation. After each story, the facilitator should check if the participants have understood the Case Study Scenario by asking open questions and repeating information where needed. These reminders may continue to be needed throughout the discussion of the sessions, and the facilitator should be on the look-out for any signs that views are being expressed because participants have misunderstood or forgotten important aspects of the Case Study Scenario.
- In addition to the case study scenarios, also make use of materials such as newspaper / magazine articles that report on e.g. celebrities who have talked about their own diagnosis of breast cancer. This can be a useful starting point for the first dFGD session.

**Outputs from this session:** By the end of the module, you should have: Generated a list of factors that would influence people's thinking about the return of individual finding; Understood/documented how these factors work as influences; Understood/documented the reasoning behind the views regarding research findings. In addition, you should make sure that people grasp the key points within the three concepts well since they will be referred back to in the second dFGD (follow up activity).

**Follow the instructions and use participatory methods to explore the issues described in the text below along with any other relevant issues that may come up.**

### 1. Case Study Scenario 1

a) ***Introducing Case Study Scenario 1***

In the next set of discussions, we would like to find out what you know and understand about our research and how we find out the things that you do. We think that there are many different reasons why people take part in our research and that they might, or might not, expect to know what we found out from our research. We want to talk to you today about how you think about research findings where blood is tested to look at genes and how they work. In talking about these things, we will ask you some

questions about if you think people should be told what is found in research, how would people like to be told, why or why not would people like to be told, and when should we tell people what was found.

To help us talk about these issues, we will give you some example scenarios from studies that are sometimes conducted in South Africa / Botswana. We will explain these to you as a story, and we will ask you to talk about what you understand from what was said and what questions you might have. So, while you are listening to the story, please try to pick out anything that stands out to you. *At the end, check understanding of the main points in this scenario.*

*Question to the group: Probe whether people know what breast cancer is.*

**Case study scenario 1: A mother participates in research that is looking at psychiatric problems when it is discovered that she has a mutation in the BRCA1 gene which increases her risk of getting breast cancer.**

**Part 1 told before asking 'GENERAL QUESTION':** In a creative way, tell a story about a mother who, after consulting with her family, participated in a research study that is looking at psychiatric problems. In the process of exploring her blood sample a researcher noticed that she carries the BRCA1 gene which means that there is a chance that she may develop breast cancer at some stage in her life. Breast cancer was not part of the focus for the study and the informed consent did not state that findings will be returned to participants.

***For the facilitator: Go to page 18 and discuss the 'General question'.***

**Part 2 told after 'GENERAL QUESTION':** The research team decides to ask the mother if she would like to know what they have found when they explored her blood sample. After talking to the researchers, the mother decides that she would like to hear what the researchers have found even if it was not related to the study. After telling the mother about the BRCA1 gene and that this increases her risk of developing breast cancer, the researchers explain that their study is not the same as a diagnostic test and that she will need to go for a test that specifically tests for the risk of breast cancer. They also explain to her that breast cancer is thought to be hereditary which means that it is caused by mutated genes passed from parent to child so she should also think about telling her 22 year old daughter to get a diagnostic test for the risk of breast cancer. The mother does not yet tell her daughter about the potential health risk but instead decides to go for the diagnostic test to find out if she is at risk for developing breast cancer, but the test is too expensive and she will not be able to pay for it.

The story is aimed to bring out the information that would be considered necessary for participants to answers questions on the how, what, when, or why on the return of individual research findings in genomics research.

*The main points that should be included in the case study scenario:*

- Individual findings from genomics research: sharing with participants;
- Findings unrelated to what is being studied;
- Research is not diagnostic;
- Findings that are clinically actionable;

- Feedback of findings for the sake of solidarity;
- Concerns about privacy or confidentiality;
- Practicalities of returning results;
- Sharing individual findings: issues related to autonomy and disrespect.

Note: To make the case study scenario more interesting, you could give the characters well known names (e.g. famous TV celebrity known to the participants) or to engage the participants you could ask them to provide these names and details of the fictional characters.

b) ***Discussion Case Study Scenario 1***

**GENERAL QUESTION**: What do you think the researchers should do about these extra results, and why?

PROMPT: What do you think about the fact that researchers had not explained about the possibility of finding this result before running tests that would show it? Why do you think they did that and in what situations would that be a reasonable or unreasonable strategy?'

**AFTER TELLING PART 2 OF CASE STUDY CONTINUE WITH THE THEMES.**

**THEME 1: Research is not diagnostic**

In the case study, the researchers told the mother that they think she might have a risk for developing breast cancer but that their test is not good enough to say this for sure because their test is not a diagnostic test. They suggest she goes for another test at a hospital and provide her with some details of where she can get this test.

- Do you think it was good enough for the researchers to suggest a place where the mother can get the right test to tell her if she is at risk for breast cancer?
- What other thing might have been right for them?
- Could the researchers have done more to help the mother find out if she is at risk for breast cancer?

  PROMPT: Should researchers pay for the test? Should they pay for the treatment?

***Look out for:*** *Responses related to the interpretation, misconception or expectation that research is diagnostic. Note whether there are expectations related to treatment but do not probe in detail about expectations of treatment as this is not at all the focus of this discussion. One of the major issues participants might raise is that the mother has now been found to have an increased risk of breast cancer and the researchers have an (arguable) responsibility to help her (straight to the duty of rescue).*

**THEME 2: Findings that are clinically actionability**

Remember from the case study that the researchers decided to tell the mother what they have found because it could affect her health but also that of her daughter if her daughter inherited the gene for breast cancer. Some people feel that they only want to know what was found by researchers who looked at their genes, if this directly affects their health, or that of a loved one, and if something can be done

to improve their health. In the case study, breast cancer is something that can be prevented and treated. Other diseases might not be preventable or treatable because they are so rare or because we do not yet know enough about how they work to make people sick.

- Thinking of this, would you want to know what was found after researchers have studied your genes even when there is no help for you? Why?

***Look out for:*** *The literature states that some participants express the desire to receive feedback on findings when the findings have direct relevance to their own health or that of a loved one. Other participants have indicated that they agreed to participate in the study to advance research and do not expect to receive feedback on individual research findings. Here we are interested to know if participants only want to know about findings that are preventable and treatable. As stated before, do not go into detail about prevention or treatment as this is not the focus of the discussion. We only want to know what participants think about receiving feedback on findings that are actionable, or would they want to know even if they can do nothing about the findings? Note that some of the points discussed will be things that could be a standard for all participants where others could be context determined (i.e. a type of prevention or treatment might not be available in a specific area due to poverty etc.).*

**THEME 3: Feedback of findings for the sake of solidarity**

Some people who have participated in research feel that they participate in research to advance research but that they don't need to get anything back for their contribution. Others feel that if participants give of themselves by allowing researchers to draw blood from them, they then also deserve to get something back. In the story that we told you about the mother, it means that the researchers should be obligated to tell her what they found after studying her genes because she agreed to participate in the study and allowed them to take blood from her.

- Thinking of this, do you think that participants should get something back to recognise their role in the research and if so what?
- In genomics research, what would be a reasonable way to recognise the gift of a sample?
- Would feeding back information relevant to the participant's health be a good way to recognise the gift of a sample?

***Look out for:*** *Ideas related to solidarity, the idea that researchers and community members should work collectively to accomplish a shared goal. This could mean that there is an expectation that if participants give of themselves, they should benefit in some way e.g. feedback of individual research findings.*

**THEME 4: Findings unrelated to what is being studied**

In the case study, you were told a story of a mother who participated in a research study that was looking at psychiatric problems, she was never told by the researchers or anyone else that they might find other problems too that are not related to psychiatric problems. The researchers then found a gene that put the mother at risk for developing breast cancer. Even though the researchers did not say that they will return any findings to the participants, and specifically said that they were looking at psychiatric

problems and not breast cancer, they decided to tell the mother what they had found and told her to go for a test that can confirm a diagnosis.

- Do you think that it was right for the researchers to tell the mother what they had found out even if it was not at all what they said they were looking for?
  PROMT: Why/ why not?
- Do participants have a right to know what researchers know about their health?
  PROMPTS: Reciprocity: information as a recognition/reward to participating in research? Duty to rescue: if the researcher knows something about me, then he has an obligation to also tell me OR to help me sort out the issue.
- How do you think the mother felt about being told that she might have a risk of developing breast cancer when no one told her that participating in this research study might give her this news?
  PROMPT: Experiences of feelings such as worry, fear, anxiety, helplessness, indifference, or gratitude?

In order to find results such as the one reported in the case study, researchers have to do a lot of work to analyse their data. This takes a lot of time and effort, and it means that they cannot spend as much time doing their actual research.

- Thinking of this, do you think that it would be good for researchers to spend time to find relevant individual results in genomics research?
  PROMPT: Genomics recruits usually >2000 people, so spending even 30 min per person means spending 1000 hrs (125 work days or half a year of a full-time person)

***Look out for:*** *Some have argued that the research team has a "duty to warn" or a "duty to rescue" the participant as he or she is in a position to prevent potentially serious harm at little or no personal cost and that the participant might otherwise not discover the condition in time to change its course. But identifying these results in datasets takes time and resources. Probe here whether and how participants think these should be weighted.*

- When do you think people should be told that genomics research could tell us something about a person's health?
  PROMPT: at consent, when something is actually found, another time?
- Should a research participant have the right to say that they do not want to know what is found out about them after their genes have been studied?
  PROMPT: Even if the informed consent stated that people might be told what is found out after their genes have been looked at, could the mother have told the researchers that she doesn't want to know what they found?

***Look out for:*** *Responses related to* <u>*autonomy*</u> *(i.e. do participants think and feel that they would have the freedom of determining their own action – whether or not they want to know or share what was fed back). Responses related to* <u>*disrespect*</u> *(i.e. "it would be a disrespectful action if research volunteers*

*are treated as conduits for generating scientific data without giving due consideration to their interest in receiving information about themselves derived from their participation in research").*[2]

**THEME 5: Concerns about privacy**

In the story that we told you, the researchers suggested that the mother tell her daughter about what was found because the breast cancer gene can be passed from a parent to their child. For this reason, the researchers advised her to tell her daughter so that she can go for a diagnostic test. If the daughter also carries this gene, she could go to the doctor frequently to check her breasts and have surgery early. That could save her life. Although this could help to keep her daughter healthy, the mother is worried that she will now have to explain why she participated in a study about psychiatric problems. The mother feels that this information is private and that she did not intend to tell people what was found from studying her genes.

- Do you think the mother has the right not to have to tell her daughter, or anyone else, about the news that she received?
- Do you think that it is wrong of the mother not to tell her daughter that she might be at risk of developing breast cancer, especially because it could affect her daughter's health too?
- Should the researchers call the daughter in and tell her what was found from studying her mother's genes?

***Look out for:*** *Issues related to confidentiality (e.g. if people would feel comfortable disclosing a potential health risk even if it might put their child's health at risk too) – when this happens find out how people would think about sharing information that is private to help someone else.*

**THEME 6: Practicalities of returning results**

In the case study, the researchers did not tell the mother that she might be told what is found after studying her genes. The mother also did not ask the researchers specifically to tell her what was found after her genes were studied.

- If genomics research were to find something, what do you think the best way would be to tell people what was found?

  PROMPTS: Telephone call or come into the clinic? Who should be allowed to know what information was found after studying a person's genes (i.e. participant only or other people also)?
- Should the researchers first ask the mother's permission to take a second sample to again look for the specific finding to make sure that they have indeed found this risk gene?

[2] From: National Academies of Sciences, Engineering, and Medicine. 2018. Returning individual research results to participants: Guidance for a new research paradigm. Washington, DC: The National Academies Press. doi: https://doi.org/10.17226/25094.

## MODULE 4: Closing the information session and next steps

**Background to this module:**

- The main aim of this module is to have a final check that all the outputs from the earlier modules have been achieved and that any outstanding issues/questions from participants have either been addressed or a way of addressing them has been agreed. This means making sure that the timing and venue of the follow up dFGD 2 are all reasonable and agreed, and that everyone is clear about what to expect.

Outputs: By the end of this module, make sure participants leave with key points of understanding and views shared (including outputs from modules 2 and 3) and looking forward to next steps (dFGD 2) with clear understanding of what this will involve.

### 1. Debrief

Make sure that participants understand that these questions and case study scenarios are all hypothetical and that the NeuroGAP / NeuroDEV / CAFGEN studies are not actually feeding back information on individual genetic findings.

### 2. Making final summaries

- Summarise views on genetics and heredity and how these factors may influence participation on research studies.
- Summarise views on the fact that research findings are not diagnostic and note how this may or may not affect trustworthiness of research findings.
- Summarise views on whether people would want to know findings (i.e. on an individual or group level), and how these findings should be communicated if they are fed back and under what circumstances.
- Highlight any other main issues shared during the information session
- Address any questions that participants might have (e.g. about IFGENERA project 2).

### 3. Planning for next meeting

- Remind the participants that the information session will be followed by two dFGDs that relate back to the issues discussed in the information session.
- Discuss and agree on dates and venues for these meetings and transport arrangements. For example, suggest possible dates/places and ask participants to choose.

### 4. Closing the information session

- Thank the participants for their willingness to take part in the information session and that you look forward to further discussion during the dFGDs.
- Each participant should receive a handout highlighting the main issues discussed during the information session. They should also be encouraged to give these points some more thought before the dFGDs and to even talk to others about these issues.
- Make transport arrangements and provide transport reimbursement before participants leave.

**MODULE 5: SECOND DELIBERATIVE FOCUS GROUP DISCUSSION**

**Background to module:**

- The aim of this module is to recall and further explore some of the answers that participants provided during the first dFGD. In this round of discussion, the interviewer should challenge participants about their views and opinions raised during the first round of questioning. For this reason, it is very important to complete a preliminary analysis of the qualitative data from dFGD 1 BEFORE dFGD 2 is undertaken so that the interviewer is sure to explore unique discussions that took place within each group during dFGD 1.
- The interviewer should also use this opportunity to introduce more complex questions and to ask counter questions that may be helpful to probe in more detail some of the answers that participants provided in the first dFGD.

*The main points that should be covered in this module:*

- Recap and explore discussion from dFGD1;
- Findings without a risk of imminent harm;
- Cost of identifying relevant results.

**Outputs from this session:** By the end of the module, you should have: probed and explored in more detail answers and views raised by participant during dFGD 1.

**Follow the instructions and use participatory methods to explore the issues described in the text below along with any other relevant issues that may come up.**

**2. Recap and explore discussion from dFGD 1**

c) ***Discussion of notes from information session and dFGD 1***

Remember in the previous meeting we talked about genes and how research can sometimes tell people things about their health after their blood has been tested. Today, we will again ask you some of the same questions from our last discussion to make sure that we have understood what you have told us but also to give you the opportunity to add anything new that you did not mention before but might have thought of in the meantime. *Consider recapping the case study scenario 1.*

*Record key issues that came up from the informational session and dFGD 1 for reference here.*
*Explore each of these issues in more detail by for example asking "Thinking back to the workshop and also adding any new ideas you may have today. You mentioned [insert statement from the information session or dFGD1]. Do the rest of you agree with that point?" For example, "When I listened to the recordings of our previous dFGD, you all seemed to agree [insert statement from the information session or dFGD1]. Is that right?"*

d) ***Further complicate topics discussed in dFGD 1***

**THEME 1: Findings without a risk of imminent or serious harm**

In the case study, the researchers decided to tell the mother that she carries the gene for a risk of developing breast cancer because this is something that could affect her health. Although it is not for sure that the mother will develop cancer, the researchers thought it would be important for her to know this news since cancer is a severe but preventable disease, so she might want to make plans to get help sooner rather than later because this influences her chances of treating and surviving the cancer.

Some researchers would say that it was not necessary or right for the researchers to tell the mother this news because she had not yet shown any signs of being sick and that the researchers had no way of knowing if she would ever get sick. In our research there are a number of genetic conditions that researchers might find.

On the piece of paper that we have given you there is a table, where each block groups some examples of the genetic conditions that may come up grouped by preventability (i.e. the likelihood that the condition will develop in that person) and severity (i.e. how sick the person might become). Let's look at the table together to make sure that everybody understands what it is showing us.

In the top left corner of the table you will see conditions that are not severe and can be prevented. This means, that if someone has one of these conditions, they cannot die from it and there is help so that they don't suffer from these conditions. Some examples of these conditions in the table are: being allergic to animals such as dogs or cats; having too little iron in your blood making you feel tired all of the time; or having kidney stones. In the bottom left of the table there are some conditions that are also not severe but that cannot be prevented by doctors or anyone else but could still be treated. Some examples of these conditions in the table are: not being able to see well and needing reading glasses; being an anxious person; or vitiligo (*show participants a picture of vitiligo and explain that it is when patches of skin on a person's body does not have any pigment and appears lighter as the rest of the skin)*. If we now look at the right side of the table, the top block shows examples of conditions that are serious problems but can also be prevented. Some of these conditions are: having a drinking problem / drinking too much and being drunk constantly which is a mental health problem; or some types of cancers like the mother in the case study. The last block at the bottom right of the table shows us some examples of conditions that are very serious and that cannot be prevented but that can be treated. Some examples of these conditions are: some psychiatric problems such as depression; learning difficulties or developmental problems such as autism where children are not able to go to mainstream schools because they are not on the same level as other children; or Alzheimer's disease which you might have seen some older people develop and they begin to be very confused or forget things or the names of family members.

- Thinking of this table and what we have just spoken about, do you think that researchers should always tell participants what they have found?
- Do you think that researchers should only tell participants if there is a chance that they will develop a disease or actually get sick?
- Do you think that researchers should tell participants about conditions that are severe and cannot be prevented but only managed to some extent?

- Do you think that researchers should tell participants about conditions that are severe but can still be prevented?
- Do you think that researchers should also tell participants about conditions that are not severe but can still be prevented?
- Do you think researchers should also tell participants about conditions that are not severe but also cannot be prevented?

***Look out for:*** *This will be a complex question to discuss in the group so really try to take some time and care to make sure that all participants understand what is being asked / discussed.*

*Some people argue that the return of individual genomic results should not be expected or be compulsory in the ethical principles for human research, except for the rare occasion when presumably reliable results are of significant clinical importance and where there is a risk of imminent or serious harm to the participant if they are not disclosed. If we are not sure that a participant will get sick, should this feedback still be given? One reason to share information is because of reciprocity, if participants gave their permission for their blood to be studied, then there can arguably be other mechanisms to achieve this same goal such as offering financial compensation for time and effort. The issue of reciprocity has been discussed in detail in Theme 2 of dFGD 2 and should not again be discussed in detail in this question. Also, do not go into a detailed discussion about benefits in return for participation as this is not the focus of this study. For this question, we are interested to further explore if participants feel that any and all feedback should be available or is it only if there is a direct and severe risk to their health.*

*For this discussion, give each participant a print out of the table below to demonstrate the four types of genomic conditions that could be reported. Again, be careful not to suggest that the current studies are reporting these conditions. This is only an example for the sake of our dFGDs.*

| | |
|---|---|
| **Not severe & preventable**<br>1. Pet dander (dog) allergy<br>2. Iron deficiency anemia<br>3. Kidney stones<br>4. Lactose intolerance<br>5. Gastroesophageal reflux disease<br>6. Reduced response to ibuprofen<br>7. Chronic mild constipation<br>8. Delayed response to local anaesthetic<br>9. Increased susceptibility to cavities | **Severe & preventable**<br>1. Alcoholism * (mental health)<br>2. Asthma<br>3. Deep vein thrombosis<br>4. Familial hypercholesterolemia<br>5. Melanoma<br>6. Peanut allergy<br>7. Types II Diabetes<br>8. Malignant hyperthermia<br>9. Childhood onset hereditary colon cancer<br>10. Aortic aneurism |
| **Not severe & non-preventable**<br>1. Attention deficit hyperactivity disorder* (learning disability)<br>2. Essential tremor<br>3. Generalized anxiety * (mental health)<br>4. Hypothyroidism<br>5. Poor vision<br>6. Seasonal allergies<br>7. Turner Syndrome<br>8. Vitiligo<br>9. Mitral valve prolapse | **Severe & non-preventable**<br>1. Autism * (developmental and learning disability)<br>2. Bipolar disorder * (mental health)<br>3. Duchenne Muscular Dystrophy<br>4. Juvenile (Type I) Diabetes<br>5. Juvenile rheumatoid arthritis<br>6. Polycystic Ovarian Syndrome<br>7. Rett Syndrome * (Childhood-onset degenerative)<br>8. Acute lymphoblastic leukemia |

| | |
|---|---|
| 10. Obstructive sleep apnea | 9. Batten disease (NCL) * (Childhood-onset degenerative)<br>10. Alzheimer's disease * (adult-onset)<br>11. Huntington's disease* (adult-onset) |

***Note:*** *This table was directly copied from Holm, I. A., Iles, B. R., Ziniel, S. I., Bacon, P. L., Savage, S. K., Christensen, K. D., … Huntington, N. L. (2015). Participant Satisfaction With a Preference-Setting Tool for the Return of Individual Research Results in Pediatric Genomic Research. Journal of Empirical Research on Human Research Ethics: JERHRE, 10(4), 414–426.*
*https://doi.org/10.1177/1556264615599620*
*This article can be read as background for the questions in theme 3.*

**THEME 2: What about children?**

In case study scenario 1 we talked about a mother who participated in the research, but we now wonder how your ideas might, or might not, differ when it is a child that participates in our research and in what way this changes what parents might, or might not, expect to know about what we found out from our research.

| |
|---|
| **Complicating case study scenario 1: A mother is asked to allow her daughter to participate in research that is looking at genes that play a role in progression of HIV infection and TB infection when it is discovered that her daughter is a carrier of the BRCA1 gene believed to be a risk factor for developing breast cancer**.<br>In a creative way, tell a story about a mother who has allowed that her daughter participate in a research study that is looking at genes that play a role in progression of HIV infection and TB infection. In the process of exploring the daughter's blood sample a researcher noticed that she carries the BRCA1 gene which means that there is a chance that she may develop breast cancer at some stage in her adult life. The researchers are not sure if they should tell the mother what they have found. The little girl might not develop breast cancer and will also not do so before she is an adult. However, if they do not tell the mother, the girl my never go for a diagnostic test when she is older which is a problem because early detection could save her life if she ever developed breast cancer.<br>*The main points that should be included in the case study scenario:*<br>➢ Ethical issues related to feedback findings that may not be actionable during childhood;<br>➢ Parents' right to know and a child's right to privacy.<br>Note: To make the case study scenario more interesting, you could give the characters well known names (e.g. famous TV celebrity known to the participants) or to engage the participants you could ask them to provide these names and details of the fictional characters. |

**Ask open ended questions to check that participants have understood these main points in the story.**

**e) Discussing the Complicated version of Case Study Scenario 1**

Researchers working on a study focusing on HIV/TB discover that one of the children in the study is a carrier of the BRCA1 gene which means that she might be at risk of developing breast cancer when she is an adult.

- Do you think the researchers have a responsibility to tell the parent(s) of a child that she carries a gene that might make her sick when she is an adult?

PROMT: Is it ok to tell parents information that will only affect the child when she is an adult, which then takes away the child's decision whether to tell her parents the news when she is an adult?

- Should the researchers tell the parents specifically what they have found or only that they think that she should go for a diagnostic test when she is an adult?
- Do you think the researchers have a responsibility to tell the parent(s) of a child that she carries a gene that might make her sick when she is an adult?
- Do you think the researchers have a responsibility to tell the parent(s) of a child that she carries a gene that might make her sick even if the parents won't be able to change anything about their child's condition?

***Look out for:*** Any discussion whether findings should be available to parents where research results may not be actionable during childhood. Probe whether participants feel it is ethically sound to tell parents information that the child might have kept private if she participated as an adult participant.
