# Supplementary material for: Do solidarity and reciprocity obligations compel African researchers to feedback individual genetic results in genomics research?
Source: BMC Med Ethics. 2020 Nov 4;21:112. doi: 10.1186/s12910-020-00549-4 (PMC7640670; doi:10.1186/s12910-020-00549-4)
Supplement: Supplementary file 2 — Additional file 2. Guide for indepth interviews. [file 12910_2020_549_MOESM2_ESM.pdf]

INDEPTH INTERVIEW GUIDE

Title of the Study: Expectations and Preferences of Parents and Adolescents Regarding Feedback of Individual Genetic Findings in a Genomic Research in Botswana

Topic Guide for IDIs with lay people who have participated in genomics research in Botswana

<u>Introduction and Purpose of the study</u>

Thank you for your willingness to participate in our in-depth interviews focusing on preferred feedback on individual genetic findings. We are very grateful to you for taking the time to participate in our research study.

This interview is a follow-up on some of the issues that were discussed in the deliberative focus group discussions that you previously participated in. The purpose of this meeting is to conduct an in-depth interview to learn more from lay individuals who participate in genomic research what, when, how, and why they think individual genetic findings should be returned to participants or their parents (guardians). This information has important implications for developing best ethical practices for genomic research.

Therefore, in our interview, we will again ask you some more questions about feedback of individual genetic findings to find out more about your thoughts and preferences.

Introduction

- Introduce self, organisation
- Introduce study
- Why am I interviewing you?
- Length of Interview
- This interview part of a study that will conducted both in Botswana and in South Africa
- Explain why recording
- Confidentiality reminder – *interview transcripts only shared with research team*
- Voluntariness reminder – *no need to answer all questions*
- Compensation and potential harms
- Any questions?
- Consent form and signing of the form

1. Feedback of results to participants

1.1. In our previous group discussions, we have talked about the different categories of genetic conditions that may be discovered during participation in genomic research. However, there are other genetic results that are not health related that may also be discovered in genomics studies.

a. Considering this, do you think genetic results that relates to an individual's paternity should be fed back?
b. Do you think genetic results that relates to an individual's ancestry should be fed back?
c. In other cases, genetic results could inform reproductive choices, do you think such genetic results should be fed back?
d. What kind of genetic information should not be fed back to participants?
    - Information that is harmful? (Examples?)
    - E.g. false paternity?
e. Do you think parents have the right to know all of their child's individual genetic results OR only those genetic test results with clear and timely direct benefit?
    - If yes, why?
    - If no, why not?
f. Sometimes, researchers may want to feedback results not because it would help the participant but because it could help their family members. For instance, this would be the case if they find that one of their male participants carries variants relating to breast cancer – knowing that wouldn't help them, but it would help their sisters and daughters. In that case, do you think those results should be fed back?

2. Severe and non-preventable

2.1. In our previous focus group discussions, participants seemed to have differing opinions with regards to feedback of results for conditions that are severe and non-preventable. There were those who wanted to receive the results and those who did not want to know the results. Considering this, how then can researchers manage these divergent views to ensure that each participants' interest is protected in returning the results?

3. Actionability

3.1. Looking at the information collected in our previous group discussions, there seem to be a high expectation of assistance and support from researchers by research participants with regard to actioning results from genomics research. Do you agree that this is the case? And why is it so?

3.2. Also, some participants indicate that whenever they go for a medical test, even for those done in research, they are always expectant of extra results. What do you think could be the reasons for this expectation?

3.3. Internationally, the consensus is that only results that are 'actionable' should be fed back. This means that some kind of intervention is available that participants can take to prevent the illness from developing. This could be something concrete – for instance, an operation to insert a device in the heart, or removing the breasts so that women don't develop cancer. It could also be something softer – for instance, regular screening, or changes in diet and exercise.

a. Do you agree with this criterion of 'actionability'? Is it right that only results that the person can do something about should be fed back?
   - E.g. Alzheimer's or Huntington's disease – ok not to feedback?

b. How do we know that something is actionable for each individual person though?
   - E.g. woman in a remote rural area may not have access to operation – still ok to feedback?

c. If something is actionable for some participants in a project, but not for others (e.g. in a different country) would it be ok to feedback information only to some people but not to others?

4. Reciprocity

4.1. Some participants in the group discussions pointed that they would expect researchers to reciprocate to them in one way or the other. What do you think are some of the reasons for participants to expect reciprocity from researchers?

5. Confidentiality issues

5.1. Again, information from the focus group discussions seems to indicate that both parents and adolescents believe that it is wrong for a parent to keep to themselves information on their breast cancer genetic results that could affect their daughter as the daughter could also be at risk of getting the breast cancer. As a result, some participants indicate that researchers should be able to notify the daughter themselves if the mother has no intentions to tell the daughter, while others think that doing so will be a breach of the mother's confidentiality and could also negatively affect the relationship between the mother and daughter. What then do you think researchers should do to balance between protecting the mother's confidentiality and the health of her daughter?

5.2. a. On the other hand, some parents feel that results for conditions that are severe and not preventable should be fed back to the parent alone and they can decide on the right time to tell the child. However, it is not clear if this applies to children of all ages, what about those that are over 18 years?

b. How about for other conditions, for example; those that are not severe and preventable? What should be the procedure for feeding back such results that relate to children?

6. Practicalities of returning results

a. According to you, who should ideally feedback individual findings from a genetic study?
   - The researcher?
   - Results to be given by a doctor?

b. Who should ideally provide participants with counselling, where needed?

c. Some participants pointed out that receiving feedback on individual genetic results could bring conflict in the family, that some family member is bewitching the participant, some parents may be in denial and take the child to traditional healers to seek help while other participants could lose hope for life and consider suicide. What do you think should be done to ensure that the above negative effects are minimised?

7. Cost of follow-up care

7.1. Even when researchers give back individual research results, the patients would still need to see a doctor to go for confirmation testing. They may also need an operation (for instance, if there is a risk of developing cardiomyopathy or breast cancer) or they need annual screening. That means there is a cost associated with knowing you have a genetic predisposition. It also mean that the information is not definite but indicates a risk.

a. Considering that there is a cost associated with receiving genetic research information, do you still think this information should be fed back? Why?

b. Who should pay for follow-up care? Why?

- Explore the responsibilities of researchers, government, research participants
- How far do the responsibilities of the researchers extend? E.g. should they only arrange a clinical appointment, should they pay for the consultation etc?
- Only be responsible for mutations that fall into the research teams' area of expertise?
- *Remember that the real interest here is understanding why, with a particular focus on reciprocity.*

8. Taking a second sample

8.1. When doing research, it is always possible that some of the tubes get mixed up, or that the results for different people get mixed up. For research, that is not a problem (the overall dataset is still the same) but it would be very bad for individuals to get the wrong results of course. Some people have proposed that the solution is to approach participants and ask them for a second sample, but this has implications for cost and logistics.

a. Re-contacting people, making appointments, taking another sample, screening the other sample – this is expensive. Who should bear the cost of this?

- Researchers, funders, the healthcare system? Why?

b. If the trade-off is between not giving results or giving results with the warning that there may have been mix-up (but without collecting a second sample), do you still think researchers should feedback individual results?

9. Consent

9.1. During the focus group discussions some participants expressed that they only have the right to know results if that was agreed upon in the beginning or for conditions that relate to what is being studied. While others indicated that, their right to know applies even where there is no prior agreement to return back the results because it's their samples that are being studied and that they have to know their health status. What do you think could be done practically to manage these divergent views so as to avoid conflicts and ensure that each participant's interest is protected?

9.2. Most studies do not seek consent for feedback of results, partly because genomics studies are already difficult to explain.

a. Do you think going forward that participants should be asked for consent? For general feedback, individual feedback?

- But what if researchers find something that signal predisposition to something quite lethal (e.g. breast cancer in women) and people said 'no I don't want to know'. Have they really considered all the issues? What should be done in such a situation?

10. Educational needs of individual genetic findings for a feedback session

10.1. Now I want to ask you to share your thoughts on the use of educational material to facilitate a feedback session.

a. How can we prepare lay individuals for the feedback on individual genetic results in genomic research?
b. What kind of information would you need to make sense of individual genetic findings in genomic research?
c. What other sources of information would be helpful for you to make sense of individual genetic findings in genomic research? [Other patients sharing their experiences? Online resources? Written materials?]

Closing Section

- We have now reached the end of this interview. Is there anything else you think is important that we have not talked about today?
- Do you have any questions about what we talked about? Is there anything you need more information about?

Thank the participant.
